# Low birthweight is associated with epigenetic age acceleration in the first 3 years of life

**DOI:** 10.1093/emph/eoad019

**Published:** 2023-06-30

**Authors:** Edward B Quinn, Chu J Hsiao, Felicien M Maisha, Connie J Mulligan

**Affiliations:** Department of Anthropology, University of Florida, Gainesville, FL 32608, USA; Genetics Institute, University of Florida, Gainesville, FL 32608, USA; Department of Anthropology, University of Florida, Gainesville, FL 32608, USA; Genetics Institute, University of Florida, Gainesville, FL 32608, USA; College of Medicine, University of Florida, Gainesville, FL 32608, USA; Department of Anthropology, University of Florida, Gainesville, FL 32608, USA; Genetics Institute, University of Florida, Gainesville, FL 32608, USA; HEAL Africa Hospital, Goma, Democratic Republic of Congo; Maisha Institute, Goma, Democratic Republic of Congo; Department of Anthropology, University of Florida, Gainesville, FL 32608, USA; Genetics Institute, University of Florida, Gainesville, FL 32608, USA

**Keywords:** aging, birthweight, developmental origins of health and disease, DNA methylation, early life adversity, epigenetic aging

## Abstract

**Background and objectives:**

The Developmental Origins of Health and Disease hypothesis posits that early life adversity is associated with poor adult health outcomes. Epidemiological evidence has supported this framework by linking low birthweight with adult health and mortality, but the mechanisms remain unclear. Accelerated epigenetic aging may be a pathway to connect early life experiences with adult health outcomes, based on associations of accelerated epigenetic aging with increased morbidity and mortality.

**Methodology:**

Sixty-seven mother-infant dyads were recruited in the eastern Democratic Republic of Congo. Birthweight data were collected at birth, and blood samples were collected at birth and follow-up visits up to age 3. DNA methylation data were generated with the Illumina MethylationEPIC array and used to estimate epigenetic age. A multilevel model was used to test for associations between birthweight and epigenetic age acceleration.

**Results:**

Chronological age was highly correlated with epigenetic age from birth to age 3 (*r* = 0.95, *p* < 2.2 × 10^−16^). Variation in epigenetic age acceleration increased over time. Birthweight, dichotomized around 2500 g, predicted epigenetic age acceleration over the first 3 years of life (*b* = −0.39, *p* = 0.005).

**Conclusions and implications:**

Our longitudinal analysis provides the first evidence for accelerated epigenetic aging that emerges between birth and age 3 and associates with low birthweight. These results suggest that early life experiences, such as low birthweight, may shape the trajectory of epigenetic aging in early childhood. Furthermore, accelerated epigenetic aging may be a pathway that links low birthweight and poor adult health outcomes.

## INTRODUCTION

The Developmental Origins of Health and Disease (DOHaD) hypothesis posits that early life experiences are associated with adult health [1]. DOHaD developed as a result of epidemiological studies that linked early life exposures, such as low birthweight, with death from coronary heart disease [2]. These early studies, and many others since, have determined that birthweight reliably predicts risk factors for chronic disease and mortality in adults [3–6]. In particular, low birthweight (<2500 g) strongly predicts all-cause mortality [7]. The damaging effects of low birthweight, indicative of exposure to *in utero* undernutrition or other stress, maybe an early determining event for later life health. However, recent evidence suggests that birthweight itself may not be causally related to adult outcomes [6, 8]. Instead, low birthweight may be correlated with prenatal shifts in physiology that are the ultimate causes of birthweight-associated increases in adult risk of death and disease [9].

Birthweight is a complex phenotype with many sources of variation, including genetic variants [10], maternal depression [11], exposure to violence [12], as well as parity, infant sex, maternal alcohol use and maternal smoking [13]. Low birthweight may be understood in terms of two models of developmental plasticity [14]. A ‘constraints’ model explains low birthweight as a result of limitations in resources for growth and development. A ‘predictive adaptive response’ model, on the other hand, explains low birthweight as a response to signals that represent future environmental conditions, such as high extrinsic mortality risk. In either case, early life signals of adversity are predicted to associate with low birthweight and other alterations in fetal physiology [9]. Low birthweight, as a biomarker for broader shifts in fetal physiology, may then predict a life history schedule characterized by accelerated development. This acceleration in development may enhance reproductive success by shortening time to sexual maturation, but may come at the cost of increased risk for chronic disease in adulthood [15]. Accelerated aging may thus link early life adversity with later life risk of morbidity and mortality.

Biological pathways linking early life experiences with adult health are thought to act in part through epigenetic pathways [1]. Epigenetics refers to modifications to DNA that do not alter the nucleotide sequence but do alter the expression of DNA. The most studied form of epigenetic modification is DNA methylation, which refers to the addition of a methyl group to a cytosine nucleotide in a cytosine-guanine context. Increasingly, researchers are using epigenetic clocks to measure biological aging using DNA methylation data [16]. These clocks estimate biological age using DNA methylation sites in the genome that are known to be associated with age [17]. These epigenetic measures of biological age show unprecedented correlations with chronological age [18, 19].

More importantly, the difference between epigenetic age and chronological age, termed epigenetic age acceleration (or deceleration), has been shown to predict the timing of puberty [20, 21], risk of chronic disease [22] and mortality [22, 23]. The association of epigenetic age acceleration with negative health outcomes is well established in adults [22], but it is unclear if age acceleration in childhood also represents a risk factor for child and adult health [24, 25]. The literature is limited but several studies report associations between epigenetic age acceleration and negative health outcomes in late childhood and early adolescence such as alterations in brain structure [26, 27], depressive symptoms [28] and internalizing problems [21]. More studies are needed to determine if epigenetic age acceleration links early life experiences such as low birthweight and altered health outcomes in adulthood.

Longitudinal studies in children using an epigenetic clock developed by Horvath [18] have shown that epigenetic age acceleration emerges over time [29–31]. Epigenetic age acceleration is dynamic in early life, but becomes increasingly stable as children approach adulthood. Such a pattern would predict low correlations of epigenetic age acceleration between time points in early childhood, somewhat stronger correlations between childhood and adolescence, and the strongest correlations between time points within adulthood. This pattern in correlations of age acceleration within individuals has been previously reported. For example, Simpkin *et al*. [32]. reported a correlation of age acceleration between birth and childhood of *r* = 0.109 and between childhood and adolescence of *r* = 0.260. Even stronger correlations of age acceleration across time points within adulthood have been reported [32, 33], suggesting that trajectories of epigenetic aging may be dynamically responsive to environmental conditions in early life and less responsive in adulthood. In addition, environmental influences appear to be a major contributor to epigenetic aging profiles in the largest twin study to date as no evidence for heritability of epigenetic aging was identified [34]. An early dynamism and low heritability of epigenetic aging suggest that the epigenetic aging trajectory may be environmentally sensitive and possibly developmentally tuned [35, 36].

In light of these findings, birthweight (used as a biomarker for prenatal exposures and a potentially damaging phenotype on its own) may be associated with epigenetic aging. However, few studies have focused on an association between birthweight and epigenetic aging in childhood. The most comparable study to ours is a longitudinal study of early life influences on epigenetic age acceleration that analyzed DNA from blood samples. Simpkin *et al*. [32]. found that birthweight was positively associated with epigenetic age acceleration at age 7, but negatively associated at age 17. In longitudinal analyses, no association was found between birthweight and epigenetic age acceleration. Additional work is needed to clarify associations between birthweight and epigenetic age acceleration, especially in early childhood.

The majority of studies on epigenetic age acceleration in children focus on middle to late childhood and adolescence; in other words, longitudinal studies in early childhood are lacking. Furthermore, children from Africa are underrepresented in studies of early life epigenetic age acceleration, which limits the genetic background and range of environmental exposures that can be investigated [37]. In this paper, we report results from a longitudinal study of epigenetic age acceleration from birth to age 3 among children born in the eastern Democratic Republic of Congo (DRC). Thus, our study addresses both of these knowledge gaps. Using low birthweight as a biomarker of adverse prenatal exposures and a potentially damaging phenotype on its own, we hypothesize that low birthweight is associated with accelerated epigenetic aging in the first 3 years of life.

## METHODOLOGY

### Study sample and recruitment

Data from this study come from an ongoing collaboration between the University of Florida (UF) and HEAL Africa Hospital in Goma, DRC that started in 2009. The eastern DRC has a history of violent conflict and high rates of sexual violence [38, 39]. Sixty-seven mother-infant dyads were recruited in the sexual violence unit of the maternity ward at HEAL Africa Hospital. Due to missing data, different analyses had different sample sizes ranging from 61 to 67 infants. Recruitment took place between July 2015 and April 2017. Follow-up visits continued until May 2019. For follow-up visits, mothers were sent reminders to bring their babies to the hospital at approximately 6 months, 1 year, 2 years and 3 years of age.

The study was approved by the Western Institutional Review Board, Olympia, WA (www.wirb.com, WIRB Project #20100993), the UF IRB (Project # IRB202001503), the University of Goma and an ethical review committee at HEAL Africa Hospital. Administrators, doctors and laboratory personnel at HEAL Africa Hospital consulted on the study and gave feedback throughout the period of the study. Women were first recruited when they arrived at HEAL Africa Hospital for delivery, although many women already knew of the study through other participating mothers. Before explaining the study, women were given the choice to move to a private setting or remain in the maternity ward—most women chose to remain in the maternity ward where they felt comfortable with other mothers. The informed consent document and all discussions about the study were conducted in Congolese Swahili, which was the primary language. Women were given the opportunity to ask questions and it was explained that their participation in the study was voluntary and they could withdraw at any time even if they initially gave consent. Once women gave consent, ethnographic interviews and validated surveys were given to the mothers, and blood samples were taken from mothers and babies within 1 day of delivery. Blood samples were also collected from babies at follow-up visits.

### Generation of DNA methylation data

DNA was extracted at the UF from whole blood collected through venipuncture. QIAmp DNA Blood Mini Kits (Qiagen) were used to extract DNA; steps 1–7 were performed at HEAL Africa Hospital to stabilize the DNA sample and kill all infectious agents prior to shipment to UF. Samples with low quantity and/or quality DNA were cleaned and concentrated following a published protocol [40]. Two hundred fifty grams of DNA were shipped to the Hussman Institute for Human Genomics, University of Miami for bisulfite conversion, hybridization to the Infinium MethylationEPIC Beadchip (Illumina), and methylation typing. Samples were randomly placed across Beadchips. At UF, raw.idat files were read into R [41] and noob background corrected [42] using the *SeSame* package [43]. Quality control of DNA methylation data, including sex checks, was performed with *meffil* [44]. Technical replicates were averaged. After removing a single DNA methylation age outlier (confirmed with a Grubbs test, *p* = 4.3 × 10^−9^), samples that failed quality control checks, and averaging replicates, 155 DNA methylation samples from 67 unique individuals were available for analysis. A custom R script to check genotype data from 59 probes on the EPIC array was used to verify that longitudinal data from baseline and follow-up samples corresponded to the same unique individual.

### Estimation of epigenetic age

Many different epigenetic clocks exist to quantify epigenetic age [16]. We used Horvath’s pan-tissue clock because it was trained on samples from people with a wide range of ages (0–100 years old), including samples at birth [18]. Horvath’s clock uses methylation at 353 CpG sites to calculate epigenetic age and has been shown to be particularly accurate in children [18]. Epigenetic age acceleration is calculated as the residual of a regression of epigenetic age on chronological age. The Horvath clock and accompanying documentation are freely available at https://dnamage.genetics.ucla.edu/home. DNA methylation age estimates were obtained using the advanced analysis option, which also estimated immune cell type proportions using previously published methods [18, 45].

### Measurement of birthweight

Birthweight was measured twice and averaged. The World Health Organization (WHO) criterion of low birthweight was used to dichotomize birthweight into <2500 g and ≥2500 g categories [46]. Two babies were high birthweight according to the WHO criterion of 4000 g (i.e. 4040 and 4080 g), which was too few individuals to create a high birthweight category so they were combined with the normal birthweight babies since their epigenetic age acceleration plots were similar. A sensitivity analysis of birthweight as a continuous variable is presented in Supplementary Materials.

### Collection of covariate data

Local members of the research team conducted semi-structured interviews with mothers in the Congolese dialect of Swahili. The interview was designed to construct a personal history and to elicit information about maternal psychosocial stressors because pilot studies had demonstrated the importance of these factors for birthweight and DNA methylation [47, 48]. Data on behaviors known to affect perinatal outcomes, such as smoking and alcohol consumption, were also collected during the interview. Interviews were conducted within 1 day of delivery.

The two maternal psychosocial stress measures used in this study, war trauma and chronic stress, have been previously described [47]. Briefly, ethnographic interview methods [49] were used to elicit salient stressors for women living in this community. War trauma captured experiences related to violence associated with living in a zone of conflict and was an unweighted sum of items such as being a refugee in the past and having family members killed (theoretical range = 0–5). Chronic stress captured day-to-day experiences such as emotional abuse, a lack of help cleaning, and unhappy marriage and was an unweighted sum (theoretical range = 0–16). Factor analyses were used to construct these scales, and both showed good internal consistency [47]. In the cohort from which our sample is drawn from, war trauma had a Cronbach’s α = 0.55 and chronic stress had a Cronbach’s α = 0.75.

Anthropometric data were collected during interviews. Parity was dichotomized to distinguish primigravida and multigravida women. Delivery method (i.e. vaginal or cesarean section) was recorded. Maternal age and infant sex were also used as covariates in analyses. Smoking cigarettes was not included as a covariate because no mothers reported smoking during pregnancy.

Gestational age is a key confounder in studies of birthweight, but is difficult to assess in low-resource settings. A recently published method used DNA methylation data to construct a gestational age epigenetic clock for newborns [50]. This gestational age epigenetic clock uses DNA methylation at 176 CpG sites to estimate gestational age, with a median absolute deviation of 3.7 days [50] and has been validated in assisted reproductive settings with known embryo transfer dates. This gestational age epigenetic clock was used to estimate gestational ages for all infants included in our study.

Immune cell type distributions in blood change with time and are thus associated with age [51]. Importantly, epigenetic age acceleration has been associated with the distribution of immune cell types [33]. These findings suggest that controlling for immune cell types is critical in studies of epigenetic aging. Immune cell type abundances and proportions were estimated as previously described [18, 45] and included in the multilevel model as covariates. Specifically, estimates of naïve CD8^+^ T cells, CD8^+^CD28^-^CD45RA^-^, plasma blasts, CD4^+^ T cells, natural killer cells, monocytes and granulocytes were included in the models.

### Statistical analyses

Two measures were used to assess the accuracy of the epigenetic clock in the analyses: the median absolute difference (MAD) and repeated measures correlation coefficient [52]. The MAD refers to the median of the absolute values of the differences between chronological age and epigenetic age. A modified Pitman’s test [53] was used to formally assess differences in the variance of DNA methylation age at different timepoints. This is a paired test so only two timepoints were compared at a time. Sample sizes are indicated for each analysis. Bias resulting from attrition was probed by testing for differences in covariates between those who had no follow-up data compared to those with at least one follow-up visit. These two groups were compared using *t*-tests for continuous variables and chi-square tests for categorical variables.

Repeated measurements require specific statistical methods to account for the lack of independence of measures from the same person. Multilevel models that nest observations within participants represent a robust method that accounts for correlated outcomes. In addition, multilevel models are robust to bias resulting from missing data [54]. A null multilevel model was fit to calculate the intraclass correlation coefficient of epigenetic age acceleration, which was used to guide the modeling strategy. The intraclass correlation coefficient was 0.79, meaning that on average the correlation between any two observations of epigenetic age acceleration within an individual was 0.79. This result suggested substantial correlation within individuals in outcome (epigenetic age acceleration), clearly indicating a need for multilevel modeling.

A multilevel model with a fixed intercept and random slope was adjusted for the covariates described above: war trauma, chronic stress, maternal body mass index (BMI), maternal age, parity, alcohol use in pregnancy, delivery method, gestational age, infant sex and cell type distribution. All analyses were completed in R [41] and α = 0.05 was used for all tests.

## RESULTS

Characteristics of mothers and infants across five time points (birth, 6 months, 1, 2 and 3 years) are reported (Table 1). Sixty-seven individuals had a total of 155 observations of DNA methylation age (average of 2.3 observations/individual). Average birthweight was 2890 g (standard deviation = 553 g). More than 25% (*n* = 17) of the infants weighed less than 2500 g at birth, that is they were low birthweight. Fifty-two percent of the infants represented at birth were female and there was a majority of female infants at all follow-up visits. On average, the gestational age of infants represented at birth was full term (39.6 weeks) and remained constant in follow-up visits. Average maternal age ranged from 17.2 to 18.6 years for infants represented at birth and all follow-up visits. First-time pregnancy (i.e. primigravida) and vaginal delivery were reported for a majority of mothers. Only a small proportion of mothers reported using alcohol during pregnancy (10.9%, *n* = 7). Maternal BMI varied between 25.2 and 25.9 for infants represented at birth and all follow-up visits. With the exception of the 6-month follow-up visit that only had eight infants, mean war trauma ranged from 1.63 to 2.00 and mean chronic stress ranged from 7.83 to 9.06, indicating fairly high levels of chronic stress. A comparison of infants with and without follow-up data found no differences in all variables (all *p* > 0.05).

**Table 1.** Descriptive statistics across time points

Variables	Birth (*N* = 64)	6 months (*N* = 8)	1 year (*N* = 35)	2 years (*N* = 32)	3 years (*N* = 16)
DNA methylation age					
Mean (SD)	0.29 (0.21)	1.24 (0.36)	2.12 (0.57)	3.43 (0.72)	5.17 (1.52)
Birthweight					
Mean (SD)	2890 (553)	2850 (546)	2920 (586)	2890 (588)	2910 (613)
Low birthweight					
<2500 g	17 (26.6%)	3 (37.5%)	10 (28.6%)	9 (28.1%)	4 (25.0%)
≥2500 g	47 (73.4%)	5 (62.5%)	25 (71.4%)	23 (71.9%)	12 (75.0%)
Sex					
Female	33 (51.6%)	6 (75.0%)	22 (62.9%)	21 (65.6%)	10 (62.5%)
Male	31 (48.4%)	2 (25.0%)	13 (37.1%)	11 (34.4%)	6 (37.5%)
Gestational age^1^ (weeks)					
Mean (SD)	39.6 (1.45)	39.3 (1.37)	39.8 (1.16)	39.7 (1.21)	39.6 (1.16)
Missing	0 (0%)	1 (12.5%)	2 (5.7%)	2 (6.3%)	0 (0%)
Maternal age					
Mean (SD)	17.2 (3.60)	18.6 (3.87)	18.5 (4.69)	18.3 (4.79)	18.5 (5.29)
Missing	3 (4.7%)	1 (12.5%)	2 (5.7%)	2 (6.3%)	0 (0%)
Parity					
Primigravida	53 (82.8%)	6 (75.0%)	26 (74.3%)	24 (75.0%)	14 (87.5%)
Multigravida	8 (12.5%)	1 (12.5%)	7 (20.0%)	6 (18.8%)	2 (12.5%)
Missing	3 (4.7%)	1 (12.5%)	2 (5.7%)	2 (6.3%)	0 (0%)
Delivery method					
Cesarean	19 (29.7%)	1 (12.5%)	10 (28.6%)	8 (25.0%)	5 (31.3%)
Vaginal	45 (70.3%)	7 (87.5%)	25 (71.4%)	24 (75.0%)	11 (68.8%)
Alcohol in pregnancy					
Alcohol	7 (10.9%)	1 (12.5%)	2 (5.7%)	2 (6.3%)	1 (6.3%)
No alcohol	54 (84.4%)	6 (75.0%)	31 (88.6%)	28 (87.5%)	15 (93.8%)
Missing	3 (4.7%)	1 (12.5%)	2 (5.7%)	2 (6.3%)	0 (0%)
Maternal BMI					
Mean (SD)	25.9 (2.83)	25.9 (2.41)	25.9 (3.09)	25.9 (3.10)	25.2 (3.19)
Missing	1 (1.6%)	0 (0%)	0 (0%)	0 (0%)	0 (0%)
War trauma					
Mean (SD)	2.00 (1.07)	1.13 (0.991)	1.71 (0.987)	1.72 (0.958)	1.63 (0.885)
Chronic stress					
Mean (SD)	7.83 (3.15)	6.38 (3.38)	7.89 (3.38)	8.22 (3.41)	9.06 (3.09)

^1^Calculated using DNA methylation data and a gestational age epigenetic clock.

SD: Standard deviation.

**Figure 1.**
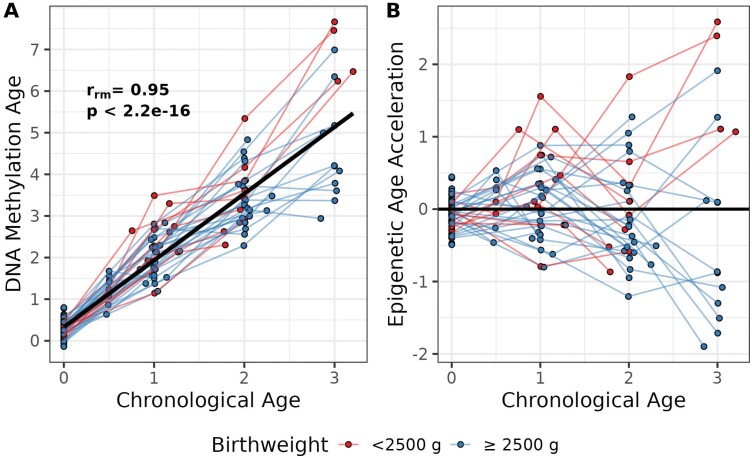
Spaghetti plot of DNA methylation age (A) and epigenetic age acceleration (B) of each infant over the first 3 years of life (*N*_individuals_ = 67, *N*_observations_ = 155). Panel A plots DNA methylation age and chronological age. A repeated measures correlation analysis revealed a high correlation between DNA methylation age and chronological age (*r*_rm_ = 0.95, *p* < 2.2e-16). Panel B plots epigenetic age acceleration (and deceleration), which is the residual from a regression of DNA methylation age on chronological age. There is an increasing variance in epigenetic age acceleration over time. Red color corresponds to birthweight <2500 g and blue color corresponds to birthweight ≥2500 g

DNA methylation age was highly correlated with chronological age in a repeated measures correlation analysis (*r*_rm_ = 0.95, *p* < 2.2e-16; Fig.1A). The MAD between DNA methylation age and chronological age became larger across all five-time points (Supplementary Table 1), ranging from a difference of 0.29 years at birth to a difference of 2.15 years at 3 years of age. Variance in DNA methylation age increased significantly between all time periods except from 1 to 2 years of age (Supplementary Table 2).

The increasing variance in DNA methylation age estimates became evident when estimates of epigenetic age acceleration were plotted (Fig. 1B). Estimates of epigenetic age acceleration were tightly clustered around zero at birth and variation in epigenetic age acceleration emerged over time. We found no evidence for differences in epigenetic age acceleration by birthweight (*p* = 0.73) or by sex (*p* = 0.21) at birth.

Multilevel models were used to determine the source(s) of variation in epigenetic age acceleration that emerged over time. There was no evidence for an association between epigenetic age acceleration and war trauma (*b* = −0.09, *p* = 0.10) or chronic stress (*b* = 0.02, *p* = 0.29) (Table 2). Several immune cell types predicted epigenetic age acceleration (Table 2). Plasma blasts (*b* = 1.32, *p* = 0.004) and naïve CD8^+^ T cells (*b* = 0.003, *p* = 0.006) were positively associated with epigenetic age acceleration and granulocytes were negatively associated with epigenetic age acceleration (*b* = −1.39, *p* = 0.01). When cell type correction was removed and three other epigenetic clocks were tested, the significance of infant age disappeared with two of the clocks, and maternal age and delivery mode became significant with two clocks (Supplementary Table 4).

**Table 2.** Multilevel model predicting epigenetic age acceleration using Horvath’s pan-tissue clock

Predictor	*b* [95% Confidence interval]
Age	0.30* [0.06, 0.54]
Birthweight (≥2500 g)	0.02 [−0.22, 0.26]
Sex (male)	−0.03 [−0.21, 0.16]
Gestational age	0.02 [−0.06, 0.10]
Maternal age	0.00 [−0.03, 0.03]
Maternal BMI	0.00 [−0.04, 0.04]
Parity (multigravida)	−0.20 [−0.52, 0.13]
Delivery mode (vaginal)	−0.02 [−0.24, 0.19]
Alcohol (none)	0.19 [−0.10, 0.49]
Naïve CD8^+^ T	0.003** [0.001, 0.006]
CD8^+^CD28^-^CD45RA^-^	0.02 [−0.02, 0.05]
Plasma blast	1.32** [0.44, 2.20]
CD4^+^ T	−0.79 [−3.23, 1.66]
Natural killer	1.67 [−1.74, 5.09]
Monocytes	−1.95 [−4.50, 0.61]
Granulocytes	−1.39* [−2.48, −0.30]
War trauma	−0.09 [−0.19, 0.02]
Chronic stress	0.02 [−0.02, 0.05]
Age × birthweight (dichotomized around 2500 g)	−0.39** [−0.64, −0.13]
*N* (observations)	141
*N* (individuals)	61
*R* ^2^ (fixed)^1^	0.24
*R* ^2^ (marginal)^2^	0.62

**p < 0.01; *p < 0.05.

^1^Fixed *R*^2^ quantifies variance explained by fixed effects alone.

^2^Marginal *R*^2^ quantifies variance explained by fixed and random effects combined.

**Figure 2.**
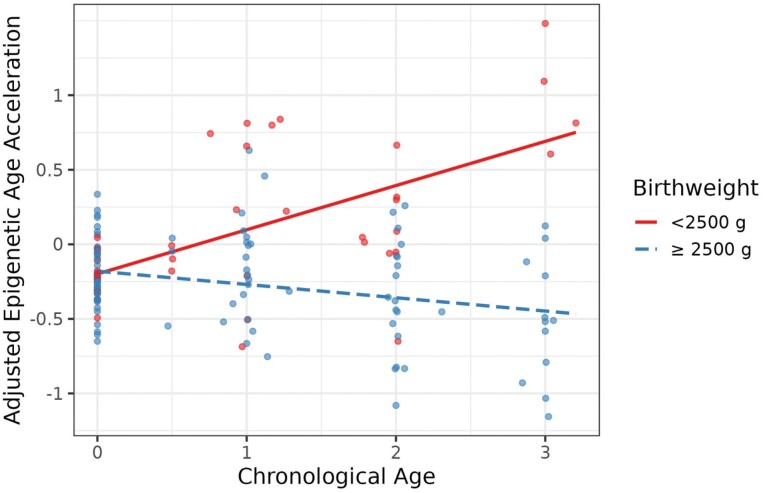
Low birthweight predicts epigenetic age acceleration. Birthweight was dichotomized at 2500 g, the WHO cutoff for low birthweight. A significant interaction (*b* = −0.39, *p* = 0.005) between age and birthweight was identified in a model adjusted for all covariates and immune cell type proportions (Table 2). The interaction effect is plotted here. A simple slopes analysis revealed that infants with birthweights <2500 g, that is low birthweight, exhibited accelerated epigenetic aging in the first 3 years of life (*b* = 0.40, *p* < 0.01), while infants with birthweights ≥2500 g showed no significant change in epigenetic age acceleration (*b* = −0.06, *p* = 0.49). *N*_individuals_ = 61, *N*_observations_ = 141. Red color corresponds to birthweight <2500 g and blue color corresponds to birthweight ≥2500 g

There was also evidence for a highly significant interaction between birthweight (dichotomized around 2500 g) and age that predicted variation in epigenetic age acceleration (*b* = −0.39, *p* = 0.005). When birthweight was analyzed as a continuous variable, the interaction effect remained significant (*b* = −0.28, *p* = 0.01; Supplementary Table 3). The interaction between birthweight and age was investigated further by plotting epigenetic age acceleration over time with birthweight dichotomized around 2500 g (Fig. 2). Low birthweight infants showed increasing epigenetic age acceleration in the first 3 years of life (*b* = 0.40, *p* < 0.01). On average, a low birthweight infant was age accelerated by 0.69 years at chronological age 3. There was no evidence for epigenetic age acceleration among infants with birthweights ≥2500 g (*b* = −0.06, *p* = 0.49).

## DISCUSSION

Early life adversity is known to impact adult health through increased risk of disease, increased mortality, and poor mental and physical health [55, 56]. Low birthweight is an example of early life adversity and is associated with increased morbidity and mortality decades later (i.e. in adulthood). The mechanism for associations between low birthweight and adult health is unclear, but our study suggests that accelerated epigenetic aging may serve as a link between low birthweight and adult health. An epigenetic signature of early life adversity and low birthweight may have evolved as a way to retain important information about the early life environment as an adaptive (or maladaptive) mechanism.

Here, we test low birthweight for associations with epigenetic age acceleration. To the best of our knowledge, this is the first longitudinal study to identify a link between low birthweight and epigenetic age acceleration between birth and age 3 (Fig. 2). Our findings suggest there may be an epigenetic link between low birthweight and biological aging.

We observed a high correlation between chronological age and epigenetic age in children from the eastern DRC (Fig. 1A). High correlations between chronological age and epigenetic age have been reported among children and adolescents in a cross-sectional study with an age range of 2–17 in the Republic of Congo [57] and among adults in other African populations [58, 59]. These data suggest that Horvath’s epigenetic clock is accurate in populations that were underrepresented in the original training data set used to construct the epigenetic clock [18], providing evidence for the transportability and validity of Horvath’s epigenetic clock [17].

In our study, epigenetic age acceleration gradually emerged over the first 3 years of life. The lowest amount of variation in epigenetic age was observed at birth, and the highest amount of variation was observed at 3 years, the oldest time point in this study (Fig. 1B and Supplementary Table 2). This pattern of findings, where increasing age in children corresponds to increasing variation in epigenetic age, has been previously reported in older children and adolescents [29–31]. Our study extends this pattern to the time between birth and age 3 for the first time. Given evidence that profiles of epigenetic age become more stable as adolescents approach adulthood [32], and throughout adulthood [33], early childhood may be a critically important time to capture the initial programming of the aging trajectory. The importance of the developmental milieu in shaping the trajectory of epigenetic age acceleration is indicated by null findings for heritability of epigenetic age in the largest twin study to date [34]. Remarkably, the twin study found that epigenetic age was not correlated within families at the start of cohabitation, but became correlated within families with greater time spent living together [34].

Our study sheds new light on predictors of the trajectory of epigenetic age acceleration in early childhood. Specifically, several immune cell types and an interaction between low birthweight and age were significant predictors of epigenetic age acceleration (Table 2). Birthweight ≥2500 g was not associated with epigenetic age acceleration or deceleration (Fig. 2). Recent studies suggest that accelerated epigenetic aging is associated with a greater risk of chronic disease and mortality [22, 23]. Consistent with low birthweight as a predictor of accelerated epigenetic aging is the strong association between low birthweight and mortality [7].

These data support a model where low birthweight is associated with accelerated epigenetic aging, which in turn is associated with a faster life history schedule and increased mortality. 

There is support for such a model in the literature on epigenetic aging in children. For example, several studies have found that accelerated epigenetic aging in childhood predicted faster pubertal development [20, 21]. Simpkin *et al*. [29]. found conflicting evidence within their study for an association between accelerated epigenetic aging and pubertal development, but they did find associations with higher fat mass at birth and greater height at age 7. Associations between greater height and epigenetic age acceleration in children have been replicated across different populations [21, 29, 57]. Collectively, these data point to epigenetic age as a marker of adaptation to faster or slower life history schedules [21, 36, 60, 61].

Findings from our study support a model where early life experiences may have an effect on the aging trajectory and associated life history events, such as reproduction and mortality [36]. This type of model construes epigenetic age acceleration as a marker of adaptation to early-life environmental signals, such as nutrient deprivation. Thus, low birthweight may be reflective of environmental signals of limitations in energetic resources, which in turn result in adaptation through a faster life history schedule. Such an adjustment in life history schedule is interpreted as adaptive under both a constraint model and a predictive adaptive response model. Integrating these two models, Berghänel *et al*. [62]. suggest that reduced maternal energetic investment in pregnancy may be adaptively counteracted by an acceleration of life history schedule by the developing fetus. Evidence for this kind of adaptation may be detected using epigenetic clocks. Sensitivity of the clock to signals of resource availability is supported by a recent *in vitro* study using human cells that found associations between epigenetic aging and markers of energy reserve, such as nutrient sensing and mitochondrial activity [63].

If epigenetic age acceleration associates with a faster life history schedule, and this faster life history schedule is predicted by low birthweight, one might expect differences in epigenetic aging predicted by birthweight to persist into adulthood. Large cohort studies in adults have found no association between birthweight and Horvath epigenetic age acceleration, but have identified associations between birthweight and other epigenetic clocks that were trained on clinical biomarkers associated with aging and telomere length [61, 64]. These results suggest that tracking the impact of low birthweight on epigenetic age acceleration may require different clocks at different ages. Several [21, 25–28], but not all [24], studies of epigenetic age acceleration in children and adolescents report associations with negative health outcomes, suggesting that epigenetic age acceleration in childhood and adolescence may be comparable to epigenetic age acceleration in adulthood, but more studies are needed to establish this equivalency. Low birthweight-associated epigenetic age acceleration could indicate an adaptive recalibration of life history scheduling in response to reduced maternal investment [62].

Our findings should be considered in the context of several limitations. We did not control for postnatal adverse exposures known to be associated with epigenetic aging in children, such as psychosocial stress and exposure to violence [57, 65–67], but we did control for prenatal exposure to violence. This is an important consideration given evidence for sensitive periods in early childhood for longitudinal effects of childhood adversity on DNA methylation patterns [66, 68]. Another limitation is our cell type estimation methods, which are based on adult references. The availability of cord blood cell type references is useful for studies at birth [69], but difficult to apply in longitudinal studies that extend beyond birth, as previously noted [29]. Strengths of the study include the longitudinal design and sampling from a theoretically important and underrepresented age and geographic group in the epigenomics literature. The epigenetic clock we used is known to be more accurate in children than other commonly used clocks [32, 70]. Answering a call for greater diversity in participants in epigenomic research [37], we describe variation in epigenetic aging among an underrepresented African population.

## CONCLUSIONS

Our study lends support for an epigenetic theory of aging, where the aging process is an outcome of early developmental processes [70]. Epigenetic age acceleration emerged within the first 3 years of life and was predicted by low birthweight. These results suggest that early life experiences, such as low birthweight, may shape the trajectory of epigenetic age acceleration in early childhood. Furthermore, accelerated epigenetic aging may be a pathway that links low birthweight and poor adult health outcomes.

## Supplementary Material

eoad019_suppl_Supplementary_Table_S1

eoad019_suppl_Supplementary_Table_S2

eoad019_suppl_Supplementary_Table_S3

eoad019_suppl_Supplementary_Table_S4

## Data Availability

DNA methylation data are available at gene expression omnibus under record GSE224573. The R script used to conduct analyses is available at github.com/edward-quinn/congo_birthweight.
