# Supplementary material for: Low birthweight is associated with epigenetic age acceleration in the first 3 years of life
Source: Evol Med Public Health. 2023 Jun 30;11(1):251–61. doi: 10.1093/emph/eoad019 (PMC10360162; doi:10.1093/emph/eoad019)
Supplement: eoad019_suppl_Supplementary_Table_S1 [file eoad019_suppl_supplementary_table_s1.docx]

| Time Point | N | Mean DNAm Age (SD) | Median Absolute Difference |
| --- | --- | --- | --- |
| Birth | 64 | 0.29 (0.21) | 0.29 |
| 6 months | 8 | 1.24 (0.36) | 0.82 |
| 1 year | 35 | 2.12 (0.57) | 0.99 |
| 2 years | 32 | 3.43 (0.72) | 1.27 |
| 3 years | 16 | 5.17 (1.52) | 2.15 |
