# Supplementary material for: Low birthweight is associated with epigenetic age acceleration in the first 3 years of life
Source: Evol Med Public Health. 2023 Jun 30;11(1):251–61. doi: 10.1093/emph/eoad019 (PMC10360162; doi:10.1093/emph/eoad019)
Supplement: eoad019_suppl_Supplementary_Table_S2 [file eoad019_suppl_supplementary_table_s2.docx]

| Time Point 1 | Variance 1 | Time Point 2 | Variance 2 | N_individuals_ | *p* |
| --- | --- | --- | --- | --- | --- |
| Birth | 0.03 | One year | 0.34 | 32 | 5.4 x 10^-5^ |
| Birth | 0.03 | Two years | 0.49 | 29 | 0.002 |
| Birth | 0.04 | Three years | 2.31 | 16 | 1.1 x 10^-5^ |
| One year | 0.33 | Two years | 0.53 | 31 | 0.25 |
| One year | 0.43 | Three years | 2.31 | 16 | 4.2 x 10^-5^ |
| Two years | 0.56 | Three years | 2.31 | 16 | 5.7 x 10^-4^ |
