# Supplementary material for: Low birthweight is associated with epigenetic age acceleration in the first 3 years of life
Source: Evol Med Public Health. 2023 Jun 30;11(1):251–61. doi: 10.1093/emph/eoad019 (PMC10360162; doi:10.1093/emph/eoad019)
Supplement: eoad019_suppl_Supplementary_Table_S3 [file eoad019_suppl_supplementary_table_s3.docx]

| Predictor | *b* [95% Confidence Interval] | |  |
| --- | --- | --- | --- |
| Age | | 0.83 * [0.20, 1.45] | |
| Birthweight (kilograms) | | 0.09 [-0.14, 0.31] |  |
| Sex (Male) | | -0.06 [-0.25, 0.13] |  |
| Gestational Age | | 0.01 [-0.07, 0.10] |  |
| Maternal Age | | 0.00 [-0.03, 0.04] |  |
| Maternal BMI | | 0.00 [-0.03, 0.04] |  |
| Parity (Multigravida) | | -0.19 [-0.53, 0.14] |  |
| Delivery Mode (Vaginal) | | -0.01 [-0.22, 0.21] |  |
| Alcohol (None) | | 0.16 [-0.14, 0.46] |  |
| naïve CD8^+^ T | | 0.00 ** [0.00, 0.00] |  |
| CD8^+^CD28^-^CD45RA^-^ | | 0.02 [-0.02, 0.05] |  |
| Plasma Blast | | 1.25 ** [0.37, 2.12] |  |
| CD4^+^ T | | -0.90 [-3.36, 1.55] |  |
| Natural Killer | | 1.78 [-1.70, 5.27] |  |
| Monocytes | | -2.27 [-4.81, 0.28] |  |
| Granulocytes | | -1.34 * [-2.43, -0.25] |  |
| War Trauma | | -0.08 [-0.18, 0.02] |  |
| Chronic Stress | | 0.02 [-0.02, 0.05] |  |
| Age x Birthweight (kilograms) | | -0.28 * [-0.49, -0.07] |  |
| N (observations) | | 141 |  |
| N (individuals) | | 61 |  |
| R^2^ (fixed)^1^ | | 0.21 |  |
| R^2^ (marginal)^2^ | | 0.63 |  |
