# Supplementary material for: Low birthweight is associated with epigenetic age acceleration in the first 3 years of life
Source: Evol Med Public Health. 2023 Jun 30;11(1):251–61. doi: 10.1093/emph/eoad019 (PMC10360162; doi:10.1093/emph/eoad019)
Supplement: eoad019_suppl_Supplementary_Table_S4 [file eoad019_suppl_supplementary_table_s4.docx]

|  | Horvath Age Acceleration  *b* [95% Confidence Interval] | Hannum Age Acceleration  *b* [95% Confidence Interval] | Intrinsic Epigenetic Age Acceleration  *b* [95% Confidence Interval] | Extrinsic Epigenetic Age Acceleration  *b* [95% Confidence Interval] |
| --- | --- | --- | --- | --- |
| Age | 0.21 [-0.02, 0.43] | 1.30 * [0.25, 2.35] | 0.20 [-0.02, 0.41] | 1.91 * [0.20, 3.62] |
| Birthweight (≥2500 grams) | -0.01 [-0.26, 0.24] | 0.92 [-0.98, 2.81] | 0.11 [-0.13, 0.36] | -0.04 [-3.17, 3.09] |
| Sex (Male) | 0.06 [-0.13, 0.25] | -0.14 [-1.40, 1.13] | -0.05 [-0.24, 0.14] | 0.21 [-1.85, 2.27] |
| Gestational Age | 0.01 [-0.08, 0.09] | 0.30 [-0.29, 0.88] | 0.01 [-0.07, 0.10] | 0.41 [-0.56, 1.38] |
| Maternal Age | 0.01 [-0.03, 0.04] | 0.02 [-0.20, 0.23] | -0.00 [-0.04, 0.03] | 0.01 [-0.34, 0.37] |
| Maternal BMI | 0.00 [-0.04, 0.04] | 0.31 * [0.04, 0.58] | -0.01 [-0.04, 0.03] | 0.49 * [0.06, 0.93] |
| Parity (Multigravida) | -0.19 [-0.53, 0.14] | -0.84 [-3.07, 1.40] | -0.14 [-0.47, 0.19] | -2.08 [-5.75, 1.59] |
| Delivery Mode (Vaginal) | 0.02 [-0.21, 0.24] | 1.69 * [0.14, 3.24] | -0.05 [-0.28, 0.17] | 2.34 [-0.20, 4.88] |
| Alcohol (None) | 0.11 [-0.20, 0.41] | 2.12 [-0.05, 4.29] | 0.12 [-0.18, 0.43] | 3.43 [-0.13, 7.00] |
| War Trauma | -0.08 [-0.18, 0.03] | 0.51 [-0.21, 1.23] | -0.08 [-0.18, 0.02] | 0.57 [-0.61, 1.74] |
| Chronic Stress | 0.01 [-0.03, 0.04] | 0.10 [-0.14, 0.34] | 0.01 [-0.03, 0.04] | 0.29 [-0.09, 0.68] |
| Age x Low Birthweight | -0.35 * [-0.62, -0.09] | -1.58 * [-2.81, -0.35] | -0.34 * [-0.59, -0.08] | -2.40 * [-4.40, -0.40] |
| N (observations) | 141 | 141 | 141 | 141 |
| N (individuals) | 61 | 61 | 61 | 61 |
| R2 (fixed)^1^ | 0.15 | 0.18 | 0.12 | 0.19 |
| R2 (total)^2^ | 0.57 | 0.19 | 0.53 | 0.19 |
|  | | | | |
